# Implication of *trans*-11,*trans*-13 conjugated linoleic acid in the development of hepatic steatosis

**DOI:** 10.1371/journal.pone.0192447

**Published:** 2018-02-01

**Authors:** Barbara D. Pachikian, Céline Druart, Emilie Catry, Laure B. Bindels, Audrey M. Neyrinck, Yvan Larondelle, Patrice D. Cani, Nathalie M. Delzenne

**Affiliations:** 1 Metabolism and Nutrition Research Group, Louvain Drug Research Institute, Université catholique de Louvain, Brussels, Belgium; 2 Institut des Sciences de la Vie, Université catholique de Louvain, Louvain-la-Neuve, Belgium; 3 Université catholique de Louvain, WELBIO, WELBIO (Walloon Excellence in Life sciences and BIOtechnology), Louvain Drug Research Institute, Metabolism and Nutrition Research Group, Brussels, Belgium; INRA, FRANCE

## Abstract

**Scope:**

Conjugated linoleic acids are linoleic acid isomers found in the diet that can also be produced through bacterial metabolism of polyunsaturated fatty acids. Our objective was to evaluate the contribution of fatty acid metabolites produced from polyunsaturated fatty acids by the gut microbiota *in vivo* to regulation of hepatic lipid metabolism and steatosis.

**Methods and results:**

In mice with depleted n-3 polyunsaturated fatty acids, we observed an accumulation of *trans*-11,*trans*-13 CLA and *cis*-9,*cis*-11 conjugated linoleic acids in the liver tissue that were associated with an increased triglyceride content and expression of lipogenic genes. We used an *in vitro* model to evaluate the impact of these two conjugated linoleic acids on hepatic lipid metabolism. In HepG2 cells, we observed that only *trans*-11,*trans*-13 conjugated linoleic acids recapitulated triglyceride accumulation and increased lipogenic gene expression, which is a phenomenon that may implicate the nuclear factors sterol regulatory element binding protein 1c (SREBP-1c) and carbohydrate-responsive element-binding protein (ChREBP).

**Conclusion:**

The *trans*-11,*trans*-13 conjugated linoleic acids can stimulate hepatic lipogenesis, which supports the conclusion that gut microbiota and related metabolites should be considered in the treatment of non-alcoholic liver disease.

## Introduction

Non-alcoholic fatty liver disease represents a major worldwide health problem with a prevalence of 20% in the general population that increases up to 70% in obese and type 2 diabetic subjects [1]. The first stage of the disease involves the accumulation of triglycerides (TG) in the liver, which is relatively benign. However, approximately 15% of non-alcoholic fatty liver disease cases evolve into hepatic inflammation (steatohepatitis) and fibrosis, and these cases can further progress to cirrhosis and hepatocellular cancer [2].

During the last decade, several studies have demonstrated that gut microbiota can regulate liver metabolism and fat storage [3, 4]. Colonization of germ-free mice with gut microbiota harvested from conventionally raised mice leads to increased body fat mass and hepatic TG content [5]. This accumulation of hepatic TG following colonization is attributed to a stimulation of *de novo* fatty acid synthesis, which is mainly controlled by a transcription factor called sterol regulatory element binding protein-1c (SREBP-1c) [5]; however, several other transcription factors, such as the Liver X Receptor, could also play a role [6, 7]. However, the molecular mechanisms explaining how gut microbes can interact with lipid metabolism in the liver remain largely unknown. The gut microbiota produce several metabolites that are able to regulate hepatic lipid metabolism, such as short chain fatty acids or secondary bile acids [8]. We and other researchers have recently found that the gut microbiota can also metabolize polyunsaturated fatty acids (PUFA) into conjugated linoleic acids (CLAs) [9–11]. CLAs represent a heterogeneous group of positional and geometric isomers of linoleic acid (LA) [12]. CLAs are mainly synthesized from free LA through the actions of bacterial isomerases in a process called biohydrogenation [13, 14]. This process was described in the rumen, but it is now recognized that human gut bacteria can perform fatty acid biohydrogenation [15–17]. The major pathway for LA biohydrogenation leads to the formation of the intermediate *cis*-9,*trans*-11 CLA. In addition to *cis*-9,*trans*-11 CLA, several other CLA isomers, such as *trans*-10,*cis*-12 CLA have been described as intermediates of LA biohydrogenation [18].

When taken as a dietary supplement, CLAs exhibit numerous beneficial health effects that are sometimes dependent on the isomer, including anti-carcinogenic, anti-inflammatory and anti-obesity effects [12, 17]. Nevertheless, these beneficial effects are, in some instances, associated with adverse effects, such as adipose tissue inflammation [19, 20] or hepatic steatosis, which occurs following stimulation of fatty acid synthesis [21–23]. However, controversies exist since some isomers also induce concomitant activation of the fatty acid oxidative pathway [24, 25]. It has been shown that dietary treatment with CLAs, specifically with *trans*-10,*cis*-12 CLA, can modify the gut microbiota composition, which is a phenomenon linked to the occurrence of steatosis [26]. However, no studies have tried to unravel which CLA moieties issued from the transformation by the gut microbiota can play a role in the regulation of fatty acid metabolism in the liver.

Feeding mice with a sunflower-diet exhibiting a low ratio in n-3/n-6 PUFA, without changing total saturated or monounsaturated fatty acids in the diet, leads to n-3 PUFA depletion and hepatic accumulation of lipids [27, 28]. This mouse model is quite interesting since it is linked to a stimulation of hepatic fatty acid synthesis, regulated by SREBP-1c, as observed in the livers of non-alcoholic fatty liver disease patients [28]. In this study, we demonstrate that two specific CLAs produced by the gut microbiota accumulate in the liver of n-3 PUFA-depleted mice. Our data from *in vivo* and *in vitro* experiments indicate that *trans-*11,*trans-*13 CLA is a key bacterial metabolite responsible for the induction of lipogenesis and ultimately hepatic lipid accumulation.

## Materials and methods

### Reagents

LA, *cis*-9,*trans*-11 CLA, *cis*-9,*cis*-11 CLA and *trans*-11,*trans*-13 CLA isomers were purchased from Larodan Fine Chemicals AB (Malmö, Sweden). FBS was purchased from Lonza (Basel, Switzerland). Dulbecco’s modified Eagle’s medium (DMEM), Trypsin-EDTA 0.25% and penicillin-streptomycin were purchased from Gibco (Inchinnan, Scotland). Fatty acid free bovine serum albumin (BSA), esiRNA human SREBF1 and esiRNA targeting RLUC were purchased from Sigma (St Louis, MO, United States).

### Animals and diets

Male C57Bl/6J mice (9 weeks old; Charles River, Brussels, Belgium) were housed in groups of 4 mice per cage at 22°C in a 12 h light/dark cycle and given free access to food and water.

After an acclimatization period of 1 week, mice were fed a control diet (CT) (D08041805, Research Diets, New Brunswick, USA) or an n-3 PUFA-depleted diet (DEF) (D08041806, Research Diets, New Brunswick, USA) for 1 month (CT-1 and DEF-1) or 3 months (CT-3 and DEF-3). The n-3 PUFA depletion was induced by replacing the soybean oil with sunflower oil (S1 Table). The total monounsaturated fatty acid and saturated fatty acid contents were similar to the CT diet, and the only difference was the n-3/n-6 PUFA ratio (S2 Table) [28]. At the end of the study period, mice fed the CT (CT-1, n = 9; CT-3, n = 4) and DEF (DEF-1, n = 9; DEF-3, n = 7) diets were anaesthetized (ketamine/xylazine i.p. at 100 and 10 mg/kg of body weight, respectively). The liver tissue was immediately clamped in liquid N_2_ and kept at -80°C until analysis. The mice were euthanized by cervical dislocation. All mouse experiments were approved by the local ethics committee for animal care of the Health Sector of the Université catholique de Louvain under the supervision of Prof. F. Lemaigre and Prof. JP Dehoux, and the housing conditions were specified by the Belgian Law of May 29, 2013, regarding the protection of laboratory animals (agreement no. LA1230314).

### Preparation of Albumin-bound CLAs

CLAs were conjugated to BSA following the modified protocol of Lee et al. [29]. A stock solution for each of the investigated CLA (3.5 mM to 14 mM) was prepared in ethanol, and aliquots were stored at −20°C. After evaporation of ethanol under nitrogen gas, 0.15 M KOH was added, and the vials were vortexed and then incubated for 1 h at 70°C. During this incubation, the vials were further vortexed for 5, 20 and 40 minutes. At the end of the incubation, filter sterilized defatted BSA in PBS (1 mM) was added to the vials to make a final CLA concentration of 5 Mm, and the vials were incubated for 1 h in a sonication bath. The pH level was adjusted to 7.0–7.5. The BSA-conjugated CLA isomers and the BSA control were stored at −20°C in tubes protected from light and evacuated under nitrogen gas.

### HepG2 cell culture and treatment

HepG2 cells (ECACC, Salisbury, UK) were cultivated at 37°C in a humidified atmosphere containing 5% CO_2_ in DMEM supplemented with 10% FBS and 1% penicillin-streptomycin. Twenty-four hours after plating, the cells were incubated for 24 h with one of the BSA-conjugated CLA at a final concentration of 100 μM or 10 μM. These concentrations were chosen based on previous results of studying the effects of CLA isomers on lipogenesis in HepG2 cells [21].

### Biochemical analyses in the liver tissue and in HepG2 cells

CLAs content was measured from liver tissue by a gas–liquid chromatograph (Focus GC, Thermo-Finnigan, Milan, Italy) equipped with a flame ionization detector after Folch extraction, as reported before [11]. For lipid content measurement in liver tissue (100 mg), lipids were extracted as described before [30]. For lipid content measurement in cultured cells (6-well plate, plated at a density of 1.3x10^6^ cells/well), lipids were extracted following the Bligh and Dyer method [31]. TG content was measured using kits from Diasys Diagnostic and Systems (Holzheim, Germany).

### Real-time quantitative PCR

Total RNA was isolated from liver tissue (50 mg) and cultured cells (6-well plate, plated at a density of 1.3x10^6^ cells/well) using the TriPure reagent (Roche, Basel, Switzerland). The cDNA was prepared by reverse transcription of 1 μg total RNA using the Kit Reverse Transcription System (Promega, Leiden, The Netherlands). Real-time qPCRs were performed with a StepOnePlus^TM^ instrument and software (Applied Biosystems, Foster City, CA, USA) using Mesa Fast qPCR MasterMix (Eurogentec, Seraing, Belgium) as described [32]. Ribosomal protein L19 (RPL19) RNA was chosen as a housekeeping gene. The primer sequences are described in the S3 Table.

### siRNA transfection

HepG2 cells were seeded at 0.5 × 10^6^ cells/well in 6-well plates. After overnight incubation at 37°C, the cells were transfected with 25 nM siRNA or Renilla luciferase negative control (RLUC). The transfection reagent DharmaFECT^TM^ (Dharmacon, Colorado, United States) was used according to the manufacturer’s instructions. Twenty-four hours after the transfection, cells were incubated for 24 h with 10 μM of *trans*-11,*trans*-13 CLA.

### Cell proliferation assay

The cell growth assay is based on metabolically active cells cleaving yellow thiazolyl blue tetrazolium bromide (MTT) to form purple formazan crystals. The formazan absorbance was measured at 570 nm, from which a background value, which was measured at 650 nm, was subtracted. Cell proliferation is expressed as a percentage of the value obtained for cells incubated with the vehicle (BSA).

### Statistical analyses

Results are presented as the mean ± SEM. Statistical significance was assessed with a Student’s *t*-test or one-way ANOVA with Tukey or Dunnett’s *post-hoc* test using GraphPad Prism version 5.00 for Windows. Associations between variables were assessed by a Pearson correlation test. p<0.05 was considered statistically significant.

## Results

### *Trans-*11,*trans*-13 and *cis-*9,*cis*-11 CLAs are the main isoforms present in the livers of mice fed a sunflower oil-based diet leading to hepatic lipid accumulation and are positively correlated with the expression of genes controlling fatty acid synthesis

We fed mice for one month or three months with a sunflower-based diet (DEF) or a soybean-based diet (CT). The sunflower-based diet had a lower n-3/n-6 PUFA ratio than the soybean-based diet leading to n-3 PUFA depletion in the liver for both DEF-1 and DEF-3 mice (Table 1). n-3 PUFA depletion also led to hepatic accumulation of monounsaturated fatty acids after only 3 months of feeding.

**Table 1:** Hepatic fatty acid profile of mice.

Composition	CT-1	DEF-1	CT-3	DEF-3
C16:0	23.87 ± 0.209	23.69 ± 0.245	23.35 ± 0.312	23.51 ± 0.719
C18:0	10.78 ± 0.642	11.07 ± 0.569	7.563 ± 0.809	6.629 ± 0.287
C20:0	0.155 ± 0.011	0.151 ± 0.007	0.375 ± 0.096	0.2998 ± 0.038
C22:0	0.034 ± 0.003	0.024 ± 0.003*	0.139 ± 0.043	0.047 ± 0.009*
C16:1 *cis*-9	2.352 ± 0.197	2.512 ± 0.241	4.109 ± 0.395	4.457 ± 0.263
C16 :1 *trans*-9	0.010 ± 0.001	0.009 ± 0.001	0.000 ± 0.000	0.000 ± 0.000
C18:1 *cis*-9	15.72 ± 1.109	17.96 ± 0.928	22.15 ± 2.145	36.91 ± 0.776*
C18 :1 *cis*-11	2.231 ± 0.129	2.728 ± 0.129*	3.495 ± 0.432	5.735 ± 0.223*
C18 :1 *trans*-9	0.033 ± 0.001	0.042 ± 0.002*	0.081 ± 0.003	0.108 ± 0.012
C18 :1 *trans*-10	0.004 ± 0.000	0.004 ± 0.000	0.000 ± 0.000	0.000 ± 0.000
C18 :1 *trans*-11	0.044 ± 0.005	0.049 ± 0.002	0.091 ± 0.020	0.043 ± 0.008*
C18:2 n-6	21.55 ± 0.599	21.52 ± 0.567	22.17 ± 0.241	10.96 ± 0.293*
C20 :4 n-6	12.81 ± 0.662	16.20 ± 0.743*	8.708 ± 1.226	9.494 ± 0.329
C18:3 n-3	0.741 ± 0.068	0.145 ± 0.008*	0.944 ± 0.054	0.217 ± 0.009*
C20 :5 n-3	0.0321 ± 0.012	0.030 ± 0.002*	0.239 ± 0.028	0.050 ± 0.005*
C22 :5 n-3	0.429 ± 0.017	0.063 ± 0.004*	0.308 ± 0.032	0.023 ± 0.002*
C22 :6 n-3	8.799 ± 0.355	3.662 ± 0.245*	6.167 ± 0.727	1.280 ± 0.071*
C18:2 *cis*-9,*trans*-11	0.035 ± 0.002	0.035 ± 0.002	0.048 ± 0.002	0.049 ± 0.001
C18:2 *trans*-10,*cis*-12	0.017 ± 0.001	0.017 ± 0.001	0.008 ± 0.001	0.003 ± 0.001*
C18:2 *cis*-9,*cis*-11	0.017 ± 0.002	0.026 ± 0.003*	0.029 ± 0.001	0.079 ± 0.004*
C18:2 *trans*-11,*trans*-13	0.015 ± 0.002	0.029 ± 0.002*	0.032 ± 0.006	0.107 ± 0.004*
C18:2 *trans*-9,*trans*-11	0.020 ± 0.003	0.036 ± 0.003*	0.005 ± 0.003	0.000 ± 0.000

Fatty acid profile (expressed as % of identified fatty acids) in mice fed a control (CT-1, n = 9; CT-3, n = 4) or an n-3 PUFA-depleted diet during one (DEF-1, n = 9) or three months (DEF-3, n = 7).

Data are the means ± SEM.

*p<0.05 versus CT mice (Student’s *t*-test).

We observed no modifications in the level of the two most studied CLAs, specifically *cis*-9,*trans*-11 CLA and *trans*-10,*cis*-12 CLA, between CT and DEF mice. However, we observed at least a two-fold increase in *cis*-9,*cis-*11 CLA and *trans*-11,*trans*-13 CLA in the livers of both DEF-1 and DEF-3 (Table 1) mice compared to CT mice, which showed the highest levels of CLAs in the livers of DEF mice. Interestingly, *cis*-9,*cis-*11 CLA and *trans*-11,*trans*-13 CLA were not detected in the DEF diet (S2 Table).

Because *cis*-9,*cis*-11 and *trans*-11,*trans-*13 CLAs were the only isoforms increased in both DEF-1 and DEF-3 mice, we decided to focus on them for the next investigation. Both DEF-1 and DEF-3 mice exhibited an increased expression of lipogenic genes in the liver. However, hepatic lipid accumulation was observed only after 3 months of depletion (Table 2).

**Table 2:** Hepatic mRNA content and TG levels in mice with depleted n-3 PUFA.

	CT-1	DEF-1	CT-3	DEF-3
SREBP-1c	1.09 ± 0.15	1.65 ± 0.19*	1.03 ± 0.15	1.66 ± 0.32
FAS	1.04 ± 0.11	1.88 ± 0.08*	1.04 ± 0.16	2.37 ± 0.21*
SCD-1	1.21 ± 0.23	1.78 ± 0.37	1.02 ± 0.12	1.41 ± 0.08*
ACCα	1.08 ± 0.16	1.73 ± 0.21*	1.02 ± 0.10	1.53 ± 0.23*
Hepatic TG	91.44 ± 5.09	93.14 ± 11.05	86.03 ± 3.29	128.4 ± 13.58*

Hepatic content in TG (expressed as nmol/mg of protein) and in sterol-regulatory element binding protein-1c (SREBP-1c), fatty acid synthase (FAS), stearoyl-CoA desaturase 1 (SCD-1) and acetyl-CoA carboxylase α (ACCα) mRNA (expressed as relative expression) in mice fed a control (CT-1, n = 9; CT-3, n = 4) or an n-3 PUFA-depleted diet for one (DEF-1, n = 9) or three months (DEF-3, n = 7).

Data are the means ± SEM.

*p<0.05 versus CT mice (Student’s *t*-test).

**Fig 1:**
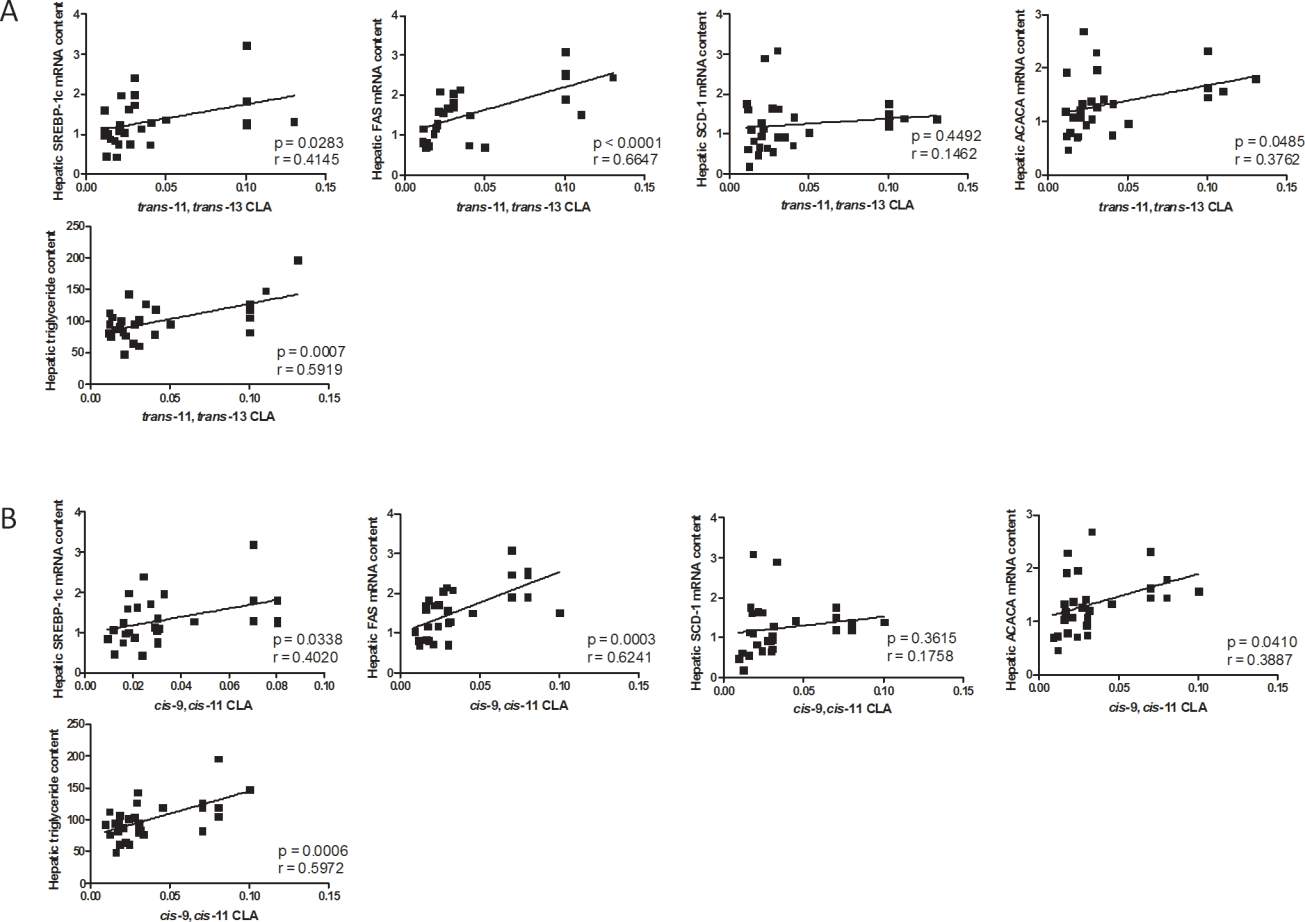
Correlations between hepatic CLAs content and mRNA expression of lipogenic genes or TG level. Correlation between the hepatic level of *cis*-9,*cis*-11 and *trans*-11,*trans*-13 CLAs and hepatic expression of genes involved in fatty acid synthesis as well as TG level in mice fed a control (CT-1, n = 9; CT-3, n = 4) or an n-3 PUFA-depleted diet for one (DEF-1, n = 9) or three months (DEF-3, n = 7). Data represent correlation coefficient; r. *p<0.05 and **p<0.01 (Pearson’s correlation test).

Both *cis*-9,*cis*-11 and *trans*-11,*trans*-13 CLAs correlated positively with the hepatic level of TG and with the expression of key genes involved in fatty acid synthesis and regulated by SREBP-1c (Fig 1).

### *Trans-*11,*trans-*13 CLA but not *cis-*9,*cis-*11 CLA supplementation increased the expression of genes involved in fatty acid synthesis and led to TG accumulation in vitro

Because *cis*-9,*cis-*11 CLA and *trans*-11,*trans*-13 CLA accumulation occurred in the livers of mice exhibiting hepatic lipid accumulation, we decided to validate the results *in vitro* using HepG2 cells to examine the effects of a supplementation with these two conjugated fatty acids on lipid metabolism. The effects were compared to results from the precursor LA and the most abundant CLA found in the liver of CT mice, specifically *cis*-9,*trans-*11 CLA (Table 1).

**Fig 2:**
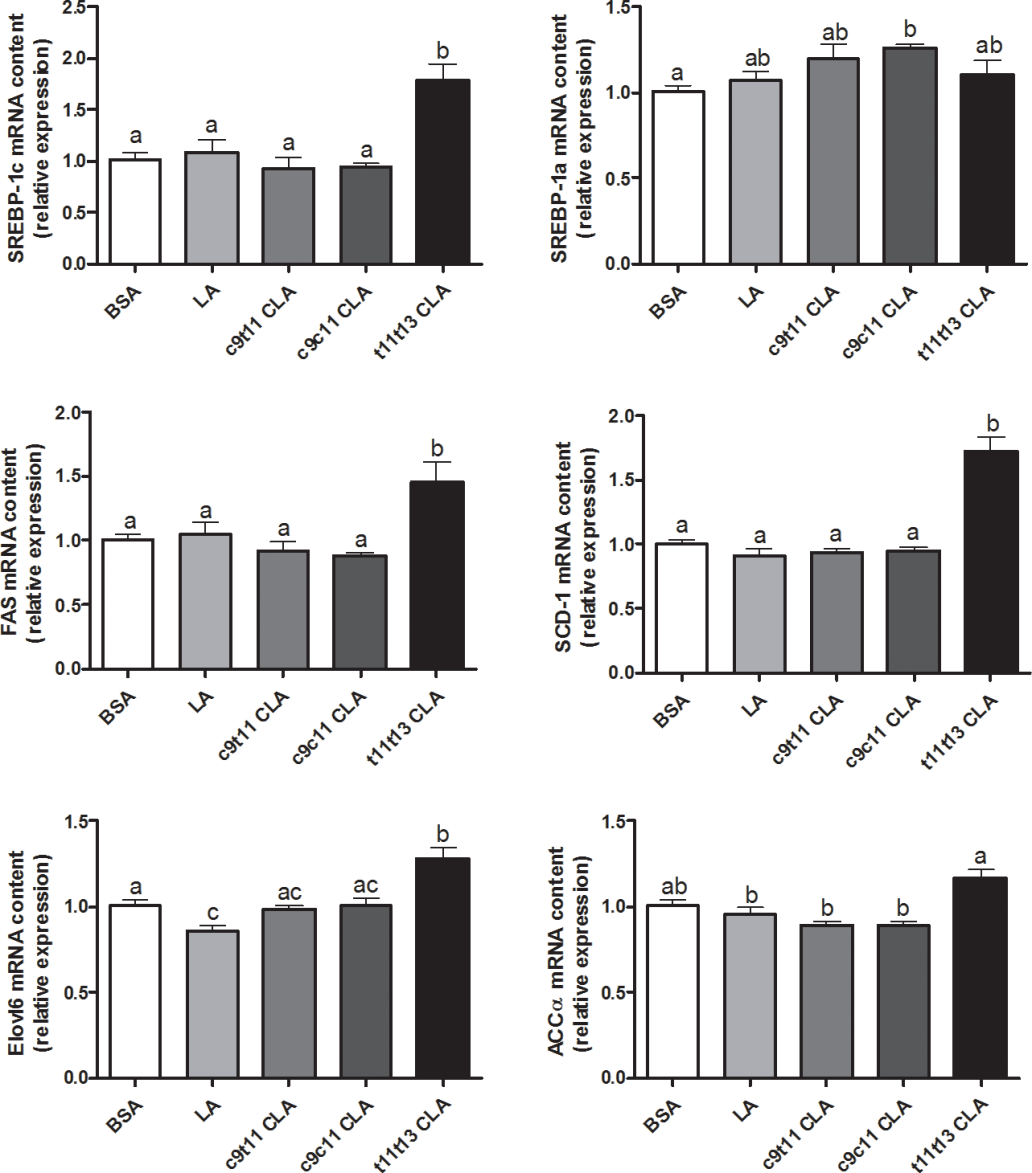
mRNA expression of lipogenic genes in HepG2 cells following CLAs treatment. HepG2 cells were incubated with bovine serum albumin (BSA) or with 10 μM of LA, *cis*-9,*trans*-11 (c9t11) CLA, *cis*-9,*cis*-11 (c9c11) CLA or *trans*-11,*trans*-13 (t11t13) CLA for 24 h. The mRNA expression of sterol-regulatory element binding protein-1c (SREBP-1c), SREBP-1a, fatty acid synthase (FAS), stearoyl-CoA desaturase 1 (SCD-1), acetyl-CoA carboxylase α (ACCα), and fatty acid elongase 6 (Elolv6) were measured by qPCR and expressed as relative expression. Data are the means ± SEM of 2 independent experiments (n = 6 to 12). Data with no common superscript letter are significantly different (p ≤ 0.05) according to the post-hoc ANOVA statistical analysis.

We used qPCR to measure the expression of key enzymes involved in the fatty acid synthesis pathway that are regulated by the transcription factor SREBP-1c (Fig 2).

**Fig 3:**
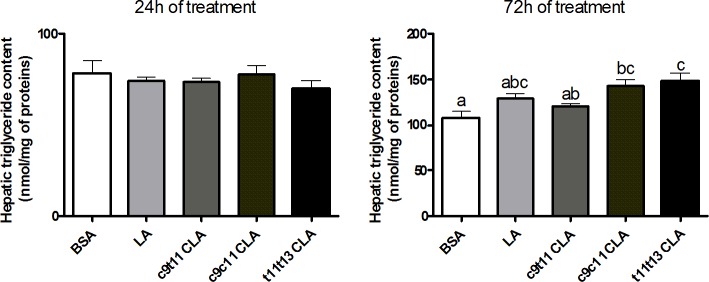
TG content in HepG2 cells following CLAs treatment. HepG2 cells were incubated with bovine serum albumin (BSA) or with 10 μM of linoleic acid (LA), *cis*-9,*trans*-11 (c9ct11) CLA, *cis*-9,*cis*-11 (c9c11) CLA or *trans*-11,*trans*-13 (t11t13) CLA for 24 h and 72 h. Hepatic TG content was expressed as nmol/mg of protein. Data are the means ± SEM of 2 independent experiments (n = 6). Data with no common superscript letter are significantly different (p ≤ 0.05) according to the post-hoc ANOVA statistical analysis.

*Trans*-11,*trans*-13 CLA increased the expression of fatty acid synthase (FAS), stearoyl-Coenzyme A desaturase 1 (SCD-1), acetyl-CoA carboxylase α (ACCα) and fatty acid elongase 6 (Elovl6) compared to the control, *cis*-9,*trans*-11 CLA and LA conditions. This result was associated with a higher expression of SREBP-1c but with SREBP-1a. The *cis*-9,*cis*-11 CLA had no effect on the expression of these genes and showed a slight stimulating effect on SREBP-1a expression. Both *trans*-11,*trans*-13 CLA and *cis*-9,*cis*-11 CLA did not modify the hepatic TG content after 24 h of supplementation, but increased TG accumulation after 72 h of incubation (Fig 3).

Interestingly, the increase in the expression of lipogenic genes was stronger following an incubation with 100 μM of *trans*-11,*trans*-13 CLA (S1 Fig), but this concentration leads to cell toxicity in the MTT test (S2 Fig).

### Possible implications of the two transcription factors SREBP-1c and Chrebp in the lipogenic effects induced by *trans-*11,*trans-*13 CLA supplementation

**Fig 4:**
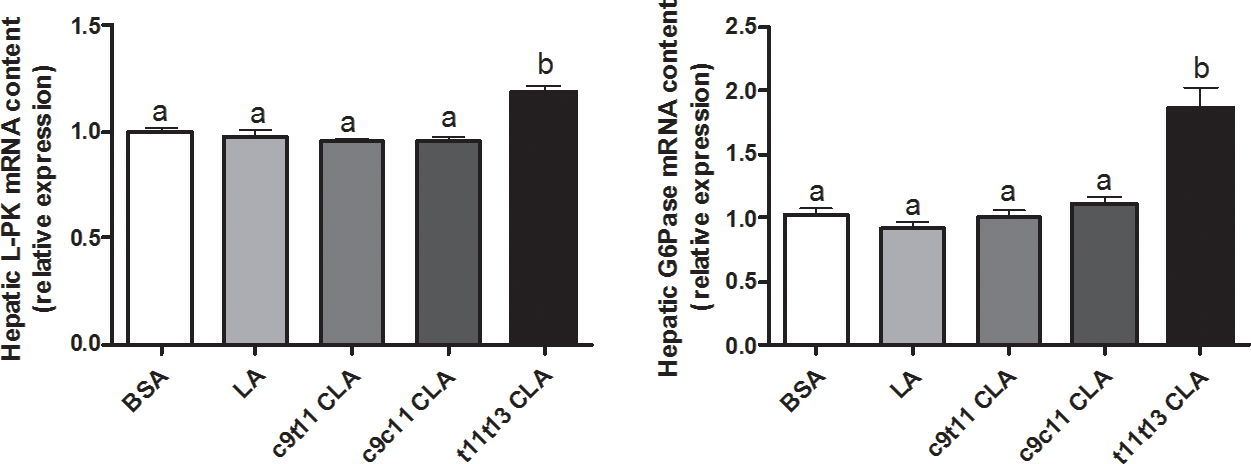
mRNA expression of carbohydrate-responsive element-binding protein (ChREBP) target genes in HepG2 cells following CLA treatment. HepG2 cells were incubated with bovine serum albumin (BSA) or with 10 μM of linoleic acid (LA), *cis*-9,*trans*-11 (c9t11) CLA, *cis*-9,*cis*-11 (c9c11) CLA or *trans*-11,*trans*-13 (t11t13) CLA for 24 h. The mRNA expression of liver pyruvate kinase (L-PK) and glucose-6 phosphatase (G6Pase) were measured by qPCR and expressed as relative expression. Data are the means ± SEM of 2 independent experiments (n = 6 to 12). Data with different superscript letters are significantly different (p ≤ 0.05) according to the post-hoc ANOVA statistical analysis.

As observed in mice with hepatic lipid accumulation, we could relate *trans*-11,*trans*-13 CLA to activation of the transcription factor SREBP-1c in HepG2 cells. To study the role of SREBP-1 in this context further, we decided to knock down SREBP-1 using SREBP-1 siRNA. Unfortunately, despite several controls using scrambled sequences, there was increased expression of lipogenic genes, which masked the effects of CLA treatment. However, we observed blocking of SREBP-1 expression by SREBP-1 siRNA, and only Elovl6 expression was decreased and was not affected by *trans*-11,*trans*-13 CLA treatment (S3 Fig). Two other transcription factors, LXR and carbohydrate-responsive element-binding protein (ChREBP), were pointed by previous microarray analysis in the liver of n-3 PUFA depleted mice for three months. Therefore, we quantify by RT-qPCR in HepG2 cells, the expression of key LXR and ChREBP target genes following *trans*-11,*trans*-13 CLA supplementation. *Trans*-11,*trans*-13 CLA supplementation led to a higher expression of L-PK and G6Pase, which are two specific ChREBP target genes (Fig 4), whereas no effect was observed on the expression of two key LXR target genes, specifically PLTP and cyp7a1, which suggests that the ChREBP pathway rather than the LXR pathway could be involved (S4 Fig).

## Discussion

Several recent studies have showed that the gut microbiota is an important factor that should be taken into account when studying non-alcoholic fatty liver disease [33, 34]. Specifically, the metabolites that it can produce appear to regulate hepatic fatty acid metabolism [35]. In our study, we found a new bacterial CLA associated with increased lipogenesis and hepatic TG accumulation in a model of nutritional depletion in n-3 PUFA (DEF mice).

Starting from LA, the major bacterial biohydrogenation pathway described by previous studies leads to the formation of *cis*-9,*trans*-11 CLA [13, 18]. In our study, the content of *cis*-9,*trans*-11 CLA was unchanged in the liver of DEF mice compared to CT mice. In contrast, two other CLA isomers seemed to be potentially relevant since the contents in *cis*-9,*cis*-11 CLA and *trans*-11,*trans*-13 CLA were almost doubled in the liver of DEF mice compared to CT mice, which became the most represented CLAs.

These two CLAs were undetectable in the DEF diet, which supports their bacterial origin. Accordingly, a recent article has shown that *trans*-11,*trans*-13 CLA was not found in the colonic content of germ-free mice, which supports the role of the gut microbiota in the accumulation of *trans*-11,*trans*-13 CLA in host tissues [10]. In our previous study [27], we described an increased level of *Roseburia* sp. in the cecal content of n-3 PUFA-depleted mice. *Roseburia* spp. are known to be involved in the production of several CLAs and could therefore be involved in the higher production of *trans*-11,*trans*-13 CLA. However, the bacteria involved in the production of *trans*-11,*trans*-13 CLA from precursors remains unexplored.

Interestingly, *cis*-9,*cis*-11 CLA and *trans*-11,*trans*-13 CLA both are both positively correlated with the hepatic TG content and with the expression of several enzymes involved in fatty acid synthesis that are regulated by the transcription factor SREBP-1c. These positive correlations were observed in the livers of both mice exhibiting TG accumulation (after three months of n-3 PUFA depletion) and before TG accumulation (one month of n-3 PUFA depletion). Specific fatty acids, such as monounsaturated fatty acids (MUFA), are also able to stimulate lipogenesis [36]. However, C18:1 *cis*-11 was the only MUFA increased in the livers of DEF mice, and the correlations with the expression of lipogenic genes and hepatic TG content were poor. Indeed, in the liver of mice fed an n-3 PUFA-depleted diet for 3 months, we observed no correlation between C18:1 *cis*-11 content and SREBP-1c (0.403, p = 0.219), FAS (0.579, p = 0.06), SCD-1 (0.343, p = 0.302) and ACCα (r = 0.291, p = 0.385). There was a significant correlation of C18:1 *cis*-11 content only with hepatic TG content (r = 0.705, p = 0.015). In the livers of mice fed the CT and DEF diets for one month, we observed a positive correlation between C18:1 *cis*-11 and FAS (r = 0.735, p = 0.0005) and ACCα (r = 0.510, p = 0.03), but not with SREBP-1c (r = 0.415, p = 0.09), SCD-1 (r = 0.457, p = 0.06) and hepatic lipid content (r = 0.215, p = 0.39).

Therefore, neither the rise in total lipid in the liver nor the rise in C18:1 *cis*-11 seems to be involved in the stimulation of lipogenesis in the livers of n-3 PUFA-depleted mice.

In HepG2 cells, we found that *trans*-11,*trans*-13 CLA was the unique isomer increasing the expression of key enzymes involved in the fatty acid synthesis pathway, specifically SCD-1, FAS, Elovl6 and ACCα after 24 h, whereas *cis*-9,*cis-*11 CLA had no effect. The stimulation of hepatic lipogenesis following *trans*-11,*trans*-13 CLA supplementation for 72 h led to an accumulation of TG. It should be mentioned that LA supplementation also increases TG content in HepG2 cells after 72 h without modifying the expression of lipogenic genes. Therefore, we could not exclude the implications of the long-term supply of fatty acids on lipid accumulation. Additionally, the accumulation of TG observed after LA supplementation was always less than the accumulation observed after *trans*-11,*trans*-13 CLA supplementation.

If the isomer *cis*-9,*trans*-11 CLA has been shown to reduce hepatic TG content in several rodent models of steatosis [37, 38], these beneficial effects have not been reported for all isomers. Indeed, *trans*-10,*cis*-12 CLA supplementation can paradoxically induce an increase in hepatic lipid content through stimulation of the lipogenic pathway and modification of fatty acid export [21, 24, 39]. This adverse effect could be attributed to a deficiency in n-3 PUFA induced by *trans*-10,*cis*-12 CLA supplementation [40]. For the first time, we described a steatogenic role for the *trans*-11,*trans*-13 CLA in liver cells.

This activation of the lipogenic pathway in the liver of mice fed for one or three months with a sunflower-based diet, which had a low n-3/n-6 PUFA ratio, was related to an activation of the transcription factor called SREBP-1c [28]. Moreover, in those mice, the hepatic content of *trans*-11,*trans*-13 CLA was positively correlated with the expression of SREBP-1c and of its target genes.

SREBP-1c belongs to a family of basic-helix-loop-helix-leucine zipper (bHLH-LZ) transcription factors and is a crucial regulator of fatty acid synthesis. There are three isoforms for SREBPs: SREBP-2 and 1a are primarily involved in the regulation of the cholesterol synthesis pathway, whereas SREBP-1c is mainly involved in the regulation of the fatty acid synthesis pathway [41]. In the liver tissue, SREBP-1c has been identified as the main isoform, while SREBP-1a is predominant in cell lines like HepG2 [31]. Consistent with the observations, *trans*-11,*trans*-13 CLA also selectively increased the expression of SREBP-1c in HepG2 cells, whereas the expression of the isoform SREBP-1a was unchanged, which suggested SREBP-1c was involved in the effect produced by *trans*-11,*trans*-13 CLA on the stimulation of fatty acid synthesis. However, after using SREBP-1 siRNA to abrogate the SREBP-1c pathway, we could not confirm the implication of SREBP-1 due to artefact problems.

Several other transcription factors are involved in the regulation of fatty acid synthesis, such as ChREBP [42], LXR, farnesoid X receptor (FXR) and PPARβ/δ [7]. LXR, regulated by oxysterols, stimulates fatty acid synthesis and is involved in the activation of the SREBP-1c pathway [6, 43]. ChREBP, regulated by glucose, stimulates lipogenesis [42], whereas FXR, regulated by bile acids, inhibits lipogenesis through the induction of the expression of the small heterodimer partner [44]. PPARβ/δ is a transcription factor which, on one hand, inhibits SREBP-1c and, on the other hand, stimulates the synthesis of monounsaturated fatty acids to the detriment of saturated fatty acids; a way to protect against lipotoxicity [7].

In a previous study, microarray analyses obtained from the liver of n-3 PUFA depleted mice for three months, in addition to the stimulation of the SREBP-1c pathway, suggested a stimulation of LXR and ChREBP pathways [28]. However, we observed no modification of the expression of key target genes reflecting the activation of any other transcription factor involved in the regulation of the fatty acid synthesis pathway, namely FXR (small heterodimer partner) or PPARβ/δ (st3gal5, Lpin2). In HepG2 cells, we investigated further the effect of *trans*-11,*trans*-13 CLA supplementation on LXR and ChREBP pathways as they also seemed activated following the microarray analyses. We observed an increased expression of two ChREBP target genes (L-PK and G6Pase) whereas PLTP and Cyp7a1 expression, two target genes of LXR, was not modified.

Therefore, *trans*-11,*trans*-13 CLA is associated with stimulation of both SREBP-1c and ChREBP pathways in both mice and HepG2 cells. However, further studies are warranted to investigate the implication of other lipogenic transcription factors.

## Conclusion

We discovered that *trans*-11,*trans*-13 CLA issued from gut microbial biohydrogenation had a stimulatory effect on lipogenesis that led to hepatic lipid accumulation by a mechanism involving the activation of nuclear factors that typically respond to insulin and carbohydrates. These results demonstrate the importance of some fatty acid metabolites produced by the gut microbiota in the regulation of gene expression in the livers of hosts as well as the potential role of the microbiota in non-alcoholic fatty liver disease.

## Supporting information

S1 Fig mRNA expression of lipogenic genes in HepG2 cells following CLAs treatment at 100 μM. HepG2 cells were incubated with bovine serum albumin (BSA) or with 100 μM of linoleic acid (LA), *cis*-9,*trans*-11 (c9t11) CLA, *cis*-9,*cis*-11 (c9c11) CLA or *trans*-11,*trans*-13 (t11t13) CLA for 24 h. The mRNA expression of fatty acid synthase (FAS), stearoyl-CoA desaturase 1 (SCD-1), acetyl-CoA carboxylase α (ACCα), fatty acid elongase 6 (Elolv6) were measured by qPCR and expressed as relative expression. Data are the means ± SEM of 7 independent experiments (n = 4 to 14). Data with no common superscript letter are significantly different (p ≤ 0.05) according to the post-hoc ANOVA statistical analysis.
(TIF)

S2 Fig HepG2 cell viability following CLAs treatment. HepG2 cells were incubated with bovine serum albumin (BSA) or with linoleic acid (LA), *cis*-9,*trans*-11 (c9t11) CLA, *cis*-9,*cis*-11 (c9c11) CLA or *trans*-11,*trans*-13 (t11t13) CLA at the indicated concentrations for 24 h. Cell viability was determined by an MTT assay and expressed as % of surviving cells compared to control cells. Data are the means ± SEM of 2 independent experiments (n = 4 to 14). * Significantly different from the BSA control condition (p ≤ 0.05) according to a Student’s *t*-test analysis.
(TIF)

S3 Fig mRNA expression of lipogenic genes following CLA treatment in HepG2 cells with a knockdown for SREBP-1. HepG2 cells were transfected with SREBP-1 siRNA. Twenty-four hours after transfection, the cells were incubated with bovine serum albumin (BSA) or with 10 μM of *trans*-11,*trans*-13 (t11t13) CLA during 24 h. The mRNA expression of sterol regulatory element binding protein-1c (SREBP-1c), SREBP-1a, fatty acid synthase (FAS), stearoyl-CoA desaturase 1 (SCD-1), acetyl-CoA carboxylase α (ACCα), fatty acid elongase 6 (Elolv6) were measured by qPCR and expressed as relative expression. Data are the means ± SEM of 2 independent experiments (n = 4 to 8). * Significantly different from the scramble (RLUC) condition (p ≤ 0.05) according to a Student’s *t*-test statistical analysis.
(TIF)

S4 Fig mRNA expression of Liver X Receptor (LXR) target genes in HepG2 cells following CLA treatment. HepG2 cells were incubated with bovine serum albumin (BSA) or with 10 μM of linoleic acid (LA), or trans-11,trans-13 (t11t13) CLA for 24 h. The mRNA expression of phospholipid transfer protein (PLTP) and CYP7A1 cytochrome P450 family 7 subfamily A member 1 (Cyp7a1) were measured by qPCR and expressed as relative expression. Data are the means ± SEM (n = 6). Data with different superscript letters are significantly different (p ≤ 0.05) according to the post-hoc ANOVA statistical analysis.
(TIF)

S1 Table Diet composition. Formulated by Research Diets. Parenthetical numbers indicate the manufacturer's diet number.
(DOCX)

S2 Table Diet fatty acid pattern. Fatty acid composition of CT and DEF diet expressed as % of total fatty acid of the diet. ND = not detectable.
(DOCX)

S3 Table The primer sequences used for real-time quantitative PCR. (DOCX)

## Abbreviations

ACCα: acetyl-CoA carboxylase α
BSA: free fatty acid bovine serum albumin
ChREBP: carbohydrate responsive element binding protein
ChREBP: carbohydrate-responsive element-binding protein
CLA: conjugated linoleic acid
Elovl6: fatty acid elongase 6
FAS: fatty acid synthase
G6Pase: glucose-6 phosphatase
LA: linoleic acid
L-PK: liver pyruvate kinase
LXR: Liver X Receptor
PUFA: polyunsaturated fatty acid
SCD-1: stearoyl-Coenzyme A desaturase 1
SREBP: sterol regulatory element binding protein
TG: triglyceride
