# Supplementary figures and images for: Implication of trans-11,trans-13 conjugated linoleic acid in the development of hepatic steatosis
Source: PLoS One. 2018 Feb 1;13(2):e0192447. doi: 10.1371/journal.pone.0192447 (PMC5794163; doi:10.1371/journal.pone.0192447)

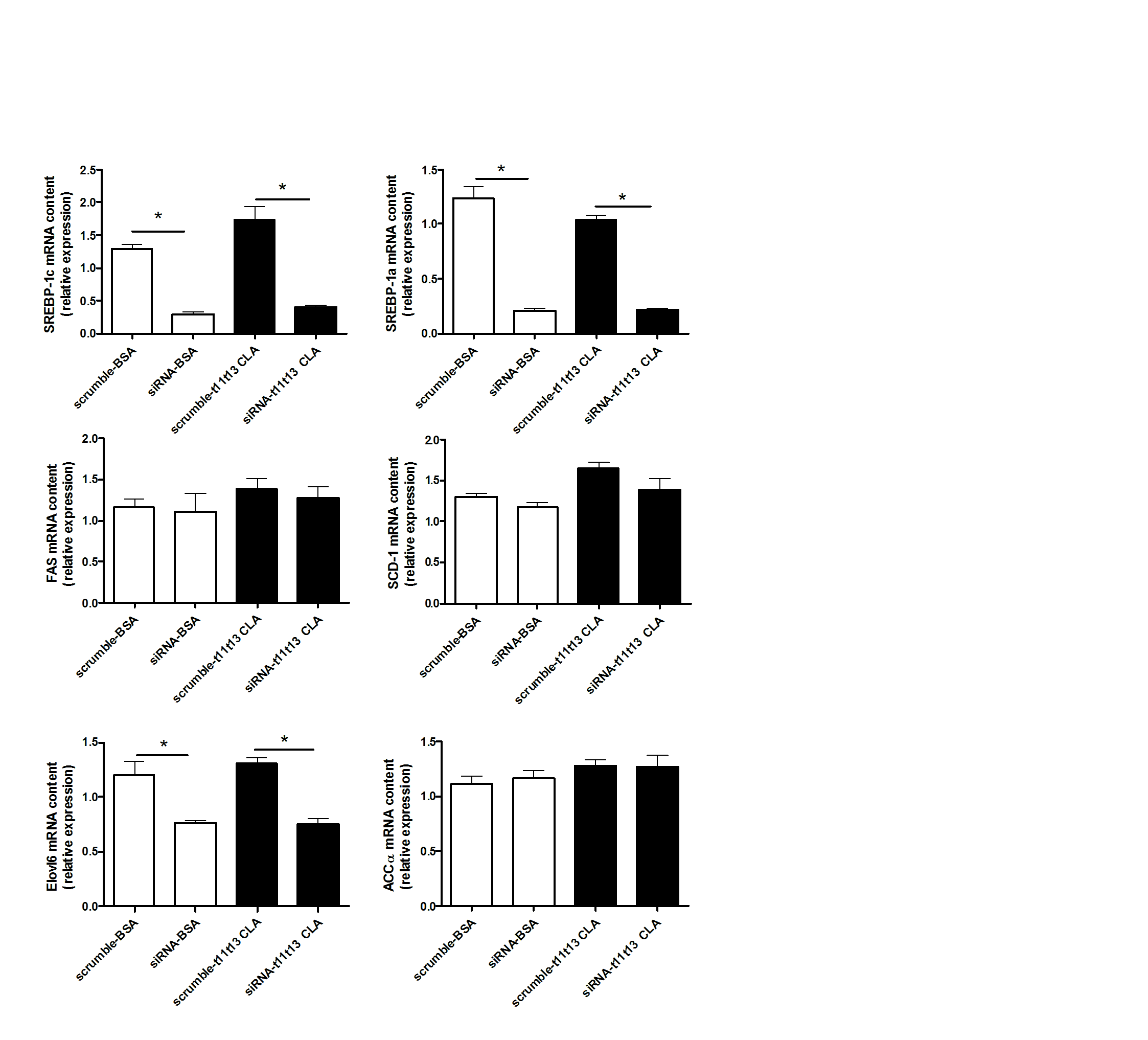

Supplement: S3 Fig — HepG2 cells were transfected with SREBP-1 siRNA. Twenty-four hours after transfection, the cells were incubated with bovine serum albumin (BSA) or with 10 μM of trans-11,trans-13 (t11t13) CLA during 24 h. The mRNA expression of sterol regulatory element binding protein-1c (SREBP-1c), SREBP-1a, fatty acid synthase (FAS), stearoyl-CoA desaturase 1 (SCD-1), acetyl-CoA carboxylase α (ACCα), fatty acid elongase 6 (Elolv6) were measured by qPCR and expressed as relative expression. Data are the means ± SEM of 2 independent experiments (n = 4 to 8). * Significantly different from the scramble (RLUC) condition (p ≤ 0.05) according to a Student’s t-test statistical analysis. (TIF) [file pone.0192447.s003.tif]

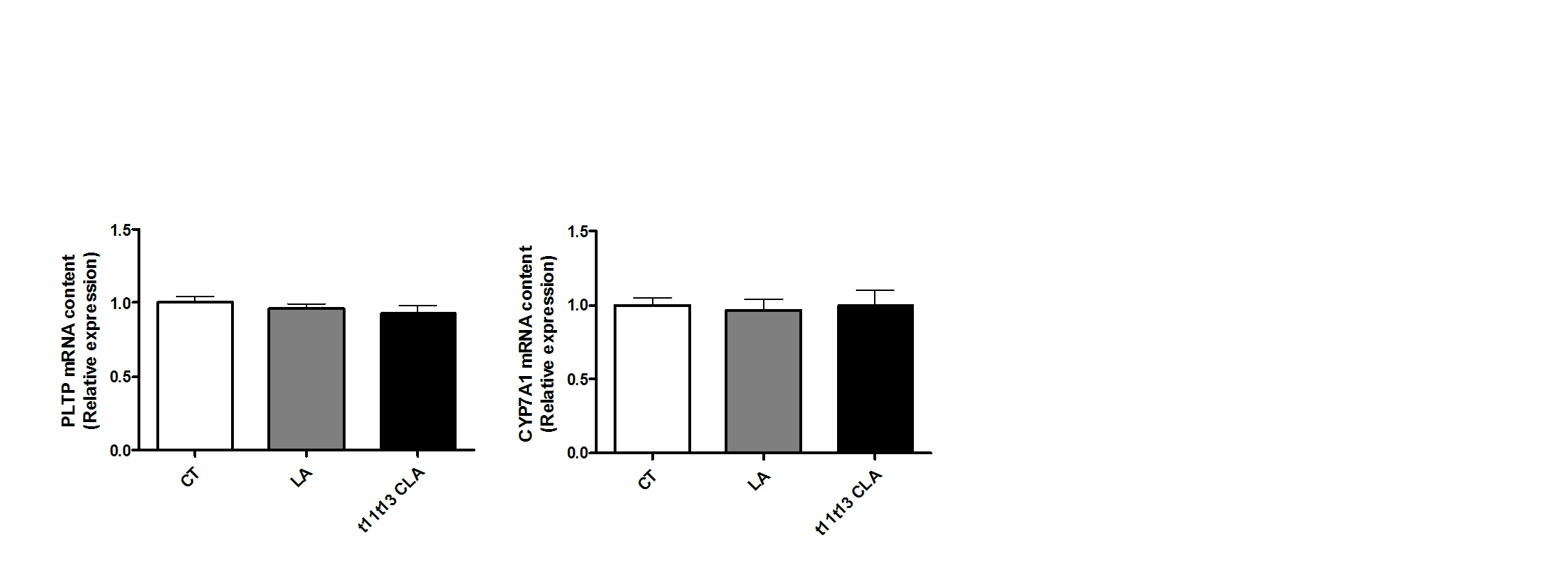

Supplement: S4 Fig — HepG2 cells were incubated with bovine serum albumin (BSA) or with 10 μM of linoleic acid (LA), or trans-11,trans-13 (t11t13) CLA for 24 h. The mRNA expression of phospholipid transfer protein (PLTP) and CYP7A1 cytochrome P450 family 7 subfamily A member 1 (Cyp7a1) were measured by qPCR and expressed as relative expression. Data are the means ± SEM (n = 6). Data with different superscript letters are significantly different (p ≤ 0.05) according to the post-hoc ANOVA statistical analysis. (TIF) [file pone.0192447.s004.tif]
