# Supplementary material for: Implication of trans-11,trans-13 conjugated linoleic acid in the development of hepatic steatosis
Source: PLoS One. 2018 Feb 1;13(2):e0192447. doi: 10.1371/journal.pone.0192447 (PMC5794163; doi:10.1371/journal.pone.0192447)
Supplement: S1 Table — Formulated by Research Diets. Parenthetical numbers indicate the manufacturer's diet number. (DOCX) [file pone.0192447.s005.docx]

| Composition | CT (D08041805) | DEF (D08041806) |
| --- | --- | --- |
| Casein (%) | 20 | 20 |
| Total carbohydrates (%)  Corn starch (%)  Sucrose (%)  Maltodextrin (%)  Cellulose (%) | 72.4  44.2  10  13.2  5 | 72.4  44.2  10  13.2  5 |
| Soybean oil (%) | 5 | 0 |
| Sunflower oil (%) | 0 | 5 |
| Mineral mix (%) | 3.5 | 3.5 |
| Vitamin mix (%) | 1 | 1 |
| n-6/n-3 PUFA | 6.9 | 127.2 |
