# Supplementary material for: Implication of trans-11,trans-13 conjugated linoleic acid in the development of hepatic steatosis
Source: PLoS One. 2018 Feb 1;13(2):e0192447. doi: 10.1371/journal.pone.0192447 (PMC5794163; doi:10.1371/journal.pone.0192447)
Supplement: S2 Table — Fatty acid composition of CT and DEF diet expressed as % of total fatty acid of the diet. ND = not detectable. (DOCX) [file pone.0192447.s006.docx]

| Composition | CT (D08041805) | DEF (D08041806) |
| --- | --- | --- |
| C16:0 | 10.614 | 7.185 |
| C18:0 | 3.809 | 4.226 |
| C20:0 | 0.302 | 0.289 |
| C22:0 | 0.320 | 0.684 |
| C16:1 *cis*-9 | 0.088 | 0.096 |
| C18:1 *cis*-9 | 21.412 | 29.698 |
| C18 :1 *cis*-11 | 1.665 | 1.285 |
| C18 :1 *trans*-9 | 0.014 | 0.015 |
| C18 :1 *trans*-10 | 0.033 | ND |
| C18 :1 *trans*-11 | 0.018 | 0.336 |
| C18:2 n-6 | 55.158 | 56.016 |
| C18:3 n-3 | 6.465 | 0.088 |
| C18:2 *cis*-9,*trans*-11 | 0.018 | 0.020 |
| C18:2 *trans*-10,*cis*-12 | 0.040 | 0.021 |
| C18:2 *cis*-9,*cis*-11 | 0.005 | ND |
| C18:2 *trans*-11,*trans*-13 | ND | ND |
| C18:2 *trans*-9,*trans*-11 | 0.040 | 0.043 |
