# Supplementary material for: Implication of trans-11,trans-13 conjugated linoleic acid in the development of hepatic steatosis
Source: PLoS One. 2018 Feb 1;13(2):e0192447. doi: 10.1371/journal.pone.0192447 (PMC5794163; doi:10.1371/journal.pone.0192447)
Supplement: S3 Table — (DOCX) [file pone.0192447.s007.docx]

| Gene | Forward (5’-3’) | Reverse (3’-5’) |
| --- | --- | --- |
| *Mouse primers:* | | |
| SREBP-1c | GATCAAAGAGGAGCCAGTGC | TAGATGGTGGCTGCTGAGTG |
| FAS | TTCCAAGACGAAAATGATGC | AATTGTGGGATCAGGAGAGC |
| SCD-1 | CCTCTTCGGGATTTTCTACTACATG | GCCGTGCCTTGTAAGTTCTGT |
| ACCα | GTTGAGACGCTGGTTTGTAGAA | GGTCCTTATTATTGTCCCAGACGTA |
| *Human primers:* | | |
| SREBP-1c | GGAGCCATGGATTGCACTTT | ATGTGGCAGGAGGTGGAGAC |
| SREBP-1a | CTGACCGACATCGAAGACAT | ATGTGGCAGGAGGTGGAGAC |
| FAS | GAGGGGACAGTGCATCAAAG | TGAGGTCCCGAGATGGTG |
| SCD-1 | CACCCAGCTGTCAAAGAGAAG | CAAGAAAGTGGCAACGAACA |
| ACCα | GAAAATCCACAATGCCAACC | CGCCAGATCCTTATTATTGTCC |
| Elovl6 | ACAATGGACCTGTCAGCAAA | GGTGATACCAGTGCAGGAAGA |
| G6Pase | TTTTGTGGTTGGGATTCTGG | CGAAGCTGAACAGGAAGAAGG |
| L-PK | GAGGAGTCTTCCCCTTGCTT | CACCACAATCACCAGGTCTC |
